# Modification of the Association between Visual Impairment and Mortality by Physical Activity: A Cohort Study among the Korean National Health Examinees

**DOI:** 10.3390/ijerph16224386

**Published:** 2019-11-10

**Authors:** Kyoung-Nam Kim, Sang Jun Park, Woosung Kim, Jungmin Joo, Haebin Kim, Kyae Hyung Kim, Ji Hoon Sohn, Yong Jin Kwon

**Affiliations:** 1 Division of Public Health and Preventive Medicine, Seoul National University Hospital, Seoul 03080, Korea; kkn002@snu.ac.kr (K.-N.K.); jungmin.alice.joo@gmail.com (J.J.); kyaehyungkim.snuh@gmail.com (K.H.K.); eliarde@naver.com (J.H.S.); 2 Department of Preventive Medicine, Seoul National University College of Medicine, Seoul 03080, Korea; sanjunpark@snu.ac.kr (S.J.P.); kimsong85@snu.ac.kr (W.K.); 3 Department of Ophthalmology, Seoul National University Bundang Hospital, Seoul National University College of Medicine, Seongnam 13620, Korea; 4 Department of Psychiatry, Kyung Hee University Hospital, Seoul 02447, Korea; haebins1001@gmail.com; 5 Department of Family Medicine, Seoul National University College of Medicine, Seoul 03080, Korea; 6 Department of Psychiatry and Behavioral Science, Seoul National University College of Medicine, Seoul 03080, Korea; 7 Department of Forensic Medicine, Seoul National University College of Medicine, Seoul 03080, Korea

**Keywords:** visual impairment, blindness, mortality, physical activity, interaction

## Abstract

The association between visual impairment and higher mortality remains unclear. In addition, evidence is lacking on the interaction between visual function and physical activity on mortality. We used data of individuals with no disability or with visual impairment among those who participated in the National Health Screening Program in Korea in 2009 or 2010. We constructed Cox proportional hazard models adjusted for potential confounders to evaluate the independent association between visual impairment and mortality. More severe visual impairment was associated with higher all-cause mortality (*p*-value for trend = 0.03) and mortality due to cardiovascular diseases (*p*-value for trend = 0.02) and that due to other diseases (*p*-value for trend = 0.01). We found an interaction on an additive scale between visual impairment and no physical activity on all-cause mortality (relative excess risk due to interaction = 1.34, 95% confidence interval: 0.37, 2.30, *p*-value = 0.01). When we stratified the study population by physical activity, the association between visual impairment and mortality was only found among individuals who did not engage in regular physical activity (*p*-value for trend = 0.01). We found an independent association between visual impairment and mortality and modification of this association by physical activity.

## 1. Introduction

Visual impairment lowers the health-related quality of life and independence in activities of daily living [1,2] and leads to adverse health outcomes such as depression and fractures, resulting in a considerable socioeconomic burden [3]. Globally, in 2015, 441.1 million people were estimated to have visual impairment. Due to worldwide population aging, the number of people with visual impairment has been sharply increasing (35.5% increase for moderate or severe visual impairment from 1990 to 2015) [4]. Visual impairment has emerged as a public health concern during the last decades.

Therefore, the effects of visual impairment on health, physical activity, chronic diseases, and mortality should be elucidated to mitigate its burden on individuals, communities, and the healthcare system. However, unlike others, the effect of visual impairment on mortality has puzzled researchers; some studies have reported an increased mortality in individuals with visual impairment [5,6,7,8,9,10,11], while others have not [12,13,14,15,16]. In addition, as visual impairment exacerbates the impact of chronic conditions on health-related quality of life [1], the association between visual impairment and mortality should be investigated considering the possible interaction between health-related conditions including physical activity, which is one of the modifiable factors affecting mortality in individuals with deteriorated vision. Physical activity has been shown to lower mortality in various populations, and, therefore, the association between visual function and mortality may differ by physical activity status. However, evidence is lacking on the interaction between visual function and physical activity with regard to mortality [5].

Therefore, in the present study, we hypothesized that visual impairment would be independently associated with increased mortality and that physical activity would modify the association between visual function and mortality. We evaluated these hypotheses using a cohort of the Korean national health examinees.

## 2. Materials and Methods

### 2.1. Study Participants

We used data from the National Health Insurance Service-National Health Screening Cohort (NHIS-HEALS) of the Republic of Korea, which has been constructed by the NHIS to enhance public research. The study protocol and detailed information of the NHIS-HEALS has been published elsewhere [17]. In brief, a random sample of the participants in the National Health Screening Program, which is free and invites all residents aged ≥40 years in the Republic of Korea at least every 2 years, constitutes this cohort (*n* = 514,866). Information on sociodemographic factors, lifestyle, past medical history, physical examinations, clinical laboratory test results, and mortality are included in the NHIS-HEALS.

In the present study, we used data of 362,285 individuals who participated in the National Health Screening Program in 2009 or 2010 because the health screening program was reorganized in 2009 and the number of survey items increased from 33 to 47 in that year. We further excluded individuals with physical (*n* = 687), auditory (*n* = 463), language (*n* = 209), intellectual (*n* = 117), and mental (*n* = 93) disabilities, brain lesions (*n* = 328), and other types of disability (including developmental, kidney, heart, and liver disabilities) (*n* = 404), leading to a final sample size of 359,984 individuals. We used information on disabilities from the National Handicapped Registry, which was provided to researchers by the NHIS-HEALS. The final study population comprised 359,523 individuals with no disabilities and 461 with visual impairment.

The Ethics Review Board of Seoul National University Hospital reviewed and approved the protocol of this study (E-1804-045-936). We conducted the present study using de-identified data (NHIS-HEALS) released to researchers for the purpose of public research.

### 2.2. Visual Impairment

In the Republic of Korea, individuals diagnosed with visual impairment by a medical doctor are registered in the National Handicapped Registry to receive disability benefits. Visual impairment is assessed by the diagnosing physicians on six grades according to severity [18]. Although individuals with visual impairment were assessed as 6 grades (grade 1 to grade 6) in the National Handicapped Registry, the NHIS-HEALS provides information on the grade of visual impairment only as “severe” (grade 1 or 2) and “mild” (grades 3–6) due to confidentiality issue. Because individuals who have a best-corrected visual acuity of ≤0.02 (20/1000) in the better-seeing eye are classified as grade 1 and those with best-corrected visual acuity of 0.02–0.04 (20/1000–20/500) in the better-seeing eye as grade 2, severe visual impairment was defined as best-corrected visual acuity ≤0.02 (20/1000) in the better-seeing eye in the present study. Meanwhile, mild visual impairment was defined as a best-corrected visual acuity of 0.06–0.2 (6/100–20/100) in the better-seeing eye, ≤0.02 (20/1000) in the worse-seeing eye, or any visual field defects (grades 3–6) in the present study.

We coded a variable for visual impairment as 0 (no visual impairment, reference), 1 (mild visual impairment), and 2 (severe visual impairment) and used it in further analyses as an independent variable.

### 2.3. Physical Activity

Information on physical activity is obtained in the NHIS-HEALS using a self-reported questionnaire inquiring on the frequency of leisure-time exercise ≥20 min, which causes rapid breathing (e.g., running, aerobics, fast cycling, or mountain climbing), corresponding to vigorous-intensity physical activity. We categorized the frequency of physical activity (day/week) as none (0 day/week), 1 or 2 days/week, 3 or 4 days/week, or ≥5 days/week and used the categorized frequency of physical activity in further analyses.

### 2.4. All-Cause and Cause-Specific Mortality

We obtained information on dates and causes of death occurring between 1 January 2010 and 31 December 2013. Statistics Korea provided the information on mortality, and the NHIS merged this information with the other data using personal identification numbers. We categorized cause of death as death caused by cardiovascular disease (defined as International Classification of Disease, 10th Revision [ICD-10] codes I20–I25, I50, and I60–I70), cancer (C00–C97), and other causes, according to a previous study [6].

### 2.5. Assumed Causal Pathway and Adjusted Covariates

**Figure 1:**
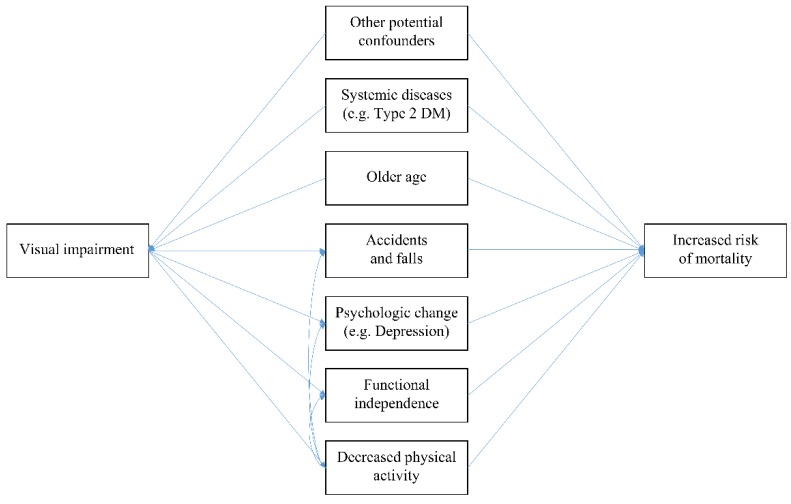
Directed acyclic graph for the association between visual impairment and mortality.

We assumed a causal pathway for the association between visual impairment and mortality as depicted in Figure 1, based on previous studies [5,6,7,8,9,12,13,14,19,20,21,22], to assess the independent association by identifying potential confounders and mediators. In brief, we selected older age, systematic diseases and conditions (body mass index, waist circumference, systolic and diastolic blood pressure, serum levels of fasting glucose, creatinine, aspartate aminotransferase, alanine aminotransferase, and gamma glutamyltransferase, and history of stroke, heart disease, hypertension, type 2 diabetes, dyslipidemia, and other diseases including cancer), income, residing area, and lifestyle factors (smoking status and alcohol consumption) as potential confounders, which could affect both visual function and mortality and possibly bias the results. After identifying the potential confounders, we adjusted them in further analyses. We did not adjust for physical activity in the main models because physical activity may mediate the association between visual function and mortality partially (Figure 1) [5,19,21] and the association between visual impairment and mortality could be underestimated when adjusting for physical activity. Detailed information on selected covariates is presented in the Supplementary Material.

### 2.6. Statistical Analysis

After visually checking that survival curves did not cross by visual function (no visual impairment, mild visual impairment, and severe visual impairment) using the Kaplan–Meier curves (Supplemental Material Figure S1), we constructed Cox proportional hazard models adjusted for potential confounders to evaluate the association between visual impairment and all-cause mortality. We also constructed cause-specific proportional hazard models adjusted for the same covariates to assess the associations between visual impairment and cause-specific mortality (mortality due to cardiovascular disease, cancer, or other diseases). We estimated *p*-values for trend by treating the variable for visual impairment as a continuous variable, not as an ordinal one. We also conducted the same analyses further adjusted for physical activity (none, 1–2, 3–4, or ≥5 times/week).

We then conducted stratified analysis using the same Cox models by the household income levels (0–3 deciles as lower income level, 4–7 as middle income level, and 8–10 as higher income level), sex, and body mass index (≥23 kg/m^2^ and <23 kg/m^2^) to identify vulnerable groups for decreased visual function.

We investigated whether physical activity would modify the association between visual impairment and mortality. It has been argued that interaction on an additive scale, rather than that on a multiplicative scale, is more suitable for evaluating biological interactions [23]. Therefore, we estimated a measure for additive interaction (i.e., relative excess risk due to interaction (RERI)). If the risk of certain outcome for exposure X_1_ is A and the risk of outcome for exposure X_2_ is B, then the RERI is greater than zero in case the risk of outcome noted as C is greater than A + B when simultaneously exposed to X_1_ and X_2_ (RERI = C − (A + B)).

To conduct this analysis, we re-categorized physical activity as no or yes (≥once/week) and assigned yes (≥once/week) as a reference because it has been reported that preventive factors (e.g., physical activity) should be recoded as risk factors to adequately estimate measures for additive interaction [24]. We also re-categorized visual impairment as no or yes (collapsed the categories for severe and mild visual impairment) and estimated the RERI between visual impairment (no or yes) and physical activity (no or yes) using logistic regression models adjusted for the same covariates. We assessed the null hypothesis that RERI = 0 using the Z-test and estimated the confidence interval (CI) and *p*-value [24]. Because a RERI score >0 denotes greater risk than the sum of each main effect in the case when exposures occur simultaneously, in the present study, a RERI score >0 can be interpreted as higher mortality due to interaction compared to the additive effects of both visual impairment and no physical activity. We assessed the association between visual impairment and mortality in each stratum stratified by physical activity (none and ≥once/week).

In sensitivity analyses, we performed analyses after excluding individuals diagnosed with vision-threatening conditions such as detachment of retinal pigment epithelium (H33.0, H33.1, H33.2, H33.3, H33.4, and H33.5), central retinal artery occlusion (H34.1), degeneration of the macula and posterior pole (H35.3), optic neuropathy (H46), visual disturbances (H53.0, H53.2, H53.2, H53.3, H53.4, H53.5, H53.6, H53.8, and H53.9), and visual impairment (H54.0, H54.1, H54.2, H54.3, H54.4, H54.6, and H54.9) between 2002 and 2010 (*n* = 25,782) among those classified in the no visual impairment group. Finally, we conducted analyses further including individuals with physical, auditory, language, intellectual, and mental disabilities, brain lesions, and other types of disability classified in the no visual impairment group.

We performed the analyses using SAS version 9.4 (SAS Institute Inc., Cary, NC, USA) and R version 3.5.2 (The Comprehensive R Archive Network, Vienna, Austria; http://cran.r-project.org).

## 3. Results

We analyzed the data of 359,523 individuals with no visual impairment, 328 with mild visual impairment, and 133 with severe visual impairment in the present study. Compared with individuals with no visual impairment, those with visual impairment were more likely to be older (71.5 years vs. 59.0 years), not exercise regularly (78.4% vs. 59.3%), and have history of stroke (6.5% vs. 1.8%), heart disease (8.4% vs. 4.7%), hypertension (51.3% vs. 36.5%), and type 2 diabetes (21.2% vs. 12.1%) (Table 1).

Because only 461 individuals with visual impairment were included compared with 359,523 with no visual impairment, we controlled for extensive aforementioned covariates in further analyses, to increase the comparability between individuals with no visual impairment and those with visual impairment. After adjusting for potential confounders, more severe visual impairment was associated with higher all-cause mortality (*p*-value for trend = 0.03) and mortality due to cardiovascular (*p*-value for trend = 0.02) and due to other diseases (*p*-value for trend = 0.01). Severe visual impairment was associated with all-cause mortality (hazard ratio [HR] = 1.90, 95% CI: 1.08, 3.35) and mortality due to cardiovascular disease (HR = 1.84, 95% CI: 1.04, 3.23) compared with no visual impairment (Table 2). When we conducted analyses further adjusted for physical activity, there were no marked changes in the results (Table 2).

**Figure 2:**
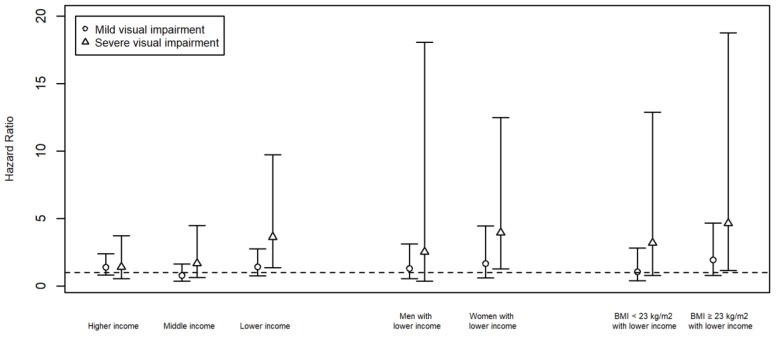
Association between visual impairment and all-cause mortality stratified by income, sex, and body mass index.

We conducted stratified analyses by household income levels, sex, and body mass index to identify vulnerable populations for visual impairment. When we stratified the analyses by household income level, more severe visual impairment was associated with higher all-cause mortality among those with lower income levels (*p*-value for trend = 0.01) but not among those with middle (*p*-value for trend = 0.79) or higher income levels (*p*-value for trend = 0.21). We found the association between severe visual impairment and all-cause mortality among those with lower income levels (HR = 3.63, 95% CI: 1.36, 9.73) but not with those with middle (HR = 1.67, 95% CI: 0.63, 4.48) or higher income levels (HR = 1.39, 95% CI: 0.52, 3.72) (Figure 2).

When we further stratified the study population with lower income levels by sex and body mass index, more severe visual impairment was associated with higher all-cause mortality among women with lower income levels (*p*-value for trend = 0.01) and individuals with lower income levels and a body mass index ≥23 kg/m^2^ (*p*-value for trend = 0.01). The association between severe visual impairment and all-cause mortality was found among women with lower income levels (HR = 3.95, 95% CI: 1.25, 12.46) but not among men with lower income levels (HR = 2.52, 95% CI: 0.35, 18.05). In addition, the association between severe visual impairment and all-cause mortality was also found among individuals with lower income levels and a body mass index ≥23 kg/m^2^ (HR = 4.64, 95% CI: 1.15, 18.74) but not among those with lower income levels and a body mass index <23 kg/m^2^ (HR = 3.18, 95% CI: 0.79, 12.87) (Figure 2). The confidence intervals were wider in these analyses, because we further stratified the lower income group by sex or body mass index and, therefore, the sample size in each stratum was smaller.

We found an interaction on an additive scale between visual impairment and no physical activity on all-cause mortality (RERI = 1.34, 95% CI: 0.37, 2.30, *p*-value = 0.01). When we stratified the study population by physical activity, an association between visual impairment and mortality was found among individuals who did not engage in regular physical activity (*p*-value for trend = 0.01), but not among those who engaged in regular physical activity (*p*-value for trend = 0.41) (Table 3).

When we excluded individuals diagnosed with possibly vision-threatening conditions among those classified in the no visual impairment group, the results were robust (Supplementary Material Table S1). The results were also robust in the analyses including individuals with physical, auditory, language, intellectual, and mental disabilities, brain lesions, and other types of disability (Supplementary Material Table S2).

## 4. Discussion

We found an independent association between visual impairment and mortality after adjusting for potential confounders, which was stronger among individuals with lower income levels. However, when we stratified the study population by physical activity, the association between visual impairment and mortality was not found among individuals who engaged in regular physical activity.

Previous studies have produced inconsistent results regarding the association between visual impairment and mortality, with some studies reporting the association [5,6,7,8,9,10,11], while other studies reporting no association [12,13,14,15,16]. This inconsistency may be due to differences in various factors, such as the sociodemographic features of the study population, the assessment method of visual function and mortality, study design, sample size, and follow-up period. Because various factors could confound or mediate the association (Figure 1), inadequate selection of covariates and specification of analytical models may also contribute to the inconsistency (e.g., adjustment of mediating factors, such as depressive symptoms and falls) [5,6,7,8,9,10,11,12,13,14,15,16]. In the present study, we performed analyses adjusted for potential confounders (older age, systemic diseases and conditions, and other potential confounders) and not mediators based on carefully assumed pathways (association mediated by increased accidents and falls [25], psychological change, such as depression [26], functional independence [27], and decreased physical activity [28]) (Figure 1). Therefore, the present study provides additional evidence for the independent association between visual impairment and mortality. However, although these pathways can be affected by social support and resource availability, and therefore, income levels, to our knowledge, no studies have reported a stronger association of visual impairment and mortality in lower income levels. Further studies are warranted to confirm the results of the present study.

Although physical activity has been associated with overall health, well-being, and decreased mortality [29,30] and reportedly individuals with visual impairment and blindness have lower physical activity levels [19,31,32], to our knowledge, only one study investigated the interaction between visual impairment and physical activity on mortality [5]. In that study, conducted with a prospective cohort in Norway, the mortality risk for both visual impairment and no physical activity was much higher than the sum of the mortality risk for only visual impairment and the mortality risk for only no physical activity. This departure from additivity was most substantial among those aged <60 years [5]. In addition, few studies have evaluated the association between physical activity and decreased mortality among individuals with visual impairment [28,33]. The results of the present study are consistent with the results of these previous studies [5,28,33]. These results highlight the importance of physical activity as a matter of concern when designing public health interventions for the visually impaired, such as developing appropriate physical activity programs and providing regular physical activity sessions through public health centers.

There are several limitations in the present study. First, although the overall prevalence of visual impairment was assessed to be 4.3% in the Republic of Korea [34], only a small proportion of individuals with visual impairment were included in the present study (359,523 with no visual impairment vs. 461 with visual impairment). However, we considered extensive factors including sociodemographic features, lifestyle, past medical history, physical examinations, and clinical laboratory test results of both participants with and those without visual impairment and controlled for the possible confounding effects of these factors in assessing the association between visual impairment and mortality. Second, instead of evaluating visual function directly during the survey, we used the information from the National Handicapped Registry [18] to identify individuals with visual impairment, leading to misclassification bias. Because diagnosis by a medical doctor and assessment of disability are needed to register in the National Handicapped Registry, it is possible that some individuals with visual impairment (especially those with mild visual impairment) were not registered in the Registry, leading to misclassification and shifting the results toward the null hypothesis. In addition, individuals with visual field defects and normal central visual acuity (e.g., patients with various diseases, such as glaucoma and retinitis pigmentosa) could have been included in the mild visual impairment group in the present study, which might also have shifted the results toward the null hypothesis. Third, the heterogeneity of the association between visual impairment and mortality by physical activity could be partly due to residual confounding of health conditions, including those pertaining to visual function, that limit physical activity, although we adjusted the analyses for extensive covariates. Fourth, the results of no association between visual impairment and mortality among individuals who engage in regular physical activity should be cautiously interpreted due to the small number of deaths, although the point estimates for the associations between visual impairment and mortality were near or less than 1, which suggest no association.

However, the present study also has some notable strengths. First, it provides new evidence for issues (e.g., interaction between visual function and physical activity on mortality and stronger association between visual impairment and mortality among those with lower income levels) that are important for implementing health policies and designing public health interventions for the visually impaired but have been investigated by a limited number of studies. Second, we considered the severity of visual impairment and assessed the dose-response association between visual function and mortality, which has not been comprehensively evaluated despite its importance in establishing causal inference [35].

## 5. Conclusions

We found an independent association between visual impairment and higher mortality adjusted for potential confounders, which was stronger among those with lower income levels. When we performed stratified analyses by physical activity, the association between visual impairment and mortality was found among individuals who did not engage in regular physical activity but not found among those who engaged in regular physical activity. Because early detection of visual impairment and effective treatment of associated problems can maximize visual function for the visually impaired, the present study suggests the importance of early detection of visual impairment and adequate treatment of associated visual problems, especially among those with lower income levels. In addition, the present study also suggests the importance of physical activity as a public health intervention for individuals with visual impairment to lower adverse health outcomes such as mortality.

## Figures and Tables

**Table 1:** Sociodemographic characteristics of the study participants by visual function (*n* = 359,984).

Characteristics	No Visual Impairment (*n* = 359,523)	Mild Visual Impairment (*n* = 328)	Severe Visual Impairment (*n* = 133)	*p*-Value ^a^
Sex				
Men	194,785 (54.2)	197 (60.1)	70 (52.6)	0.10
Women	164,738 (45.8)	131 (39.9)	63 (47.4)	
Household income (deciles)				
0–3	73,251 (20.4)	80 (24.4)	25 (18.8)	0.41
4–7	115,402 (32.1)	104 (31.7)	46 (34.6)	
8–10	170,870 (47.5)	144 (43.9)	62 (46.6)	
Body mass index (kg/m^2^)				
<18.5	7848 (2.2)	19 (5.8)	7 (5.3)	<0.01
18.5–22.9	125,809 (35.0)	114 (34.8)	48 (36.1)	
23–24.9	100,866 (28.1)	93 (28.4)	32 (24.1)	
≥25	124,894 (34.8)	102 (31.1)	46 (34.6)	
Smoking status				
Non-smoker	227,957 (64.5)	229 (70.0)	89 (68.5)	0.04
Ex-smoker	65,591 (18.5)	63 (19.3)	21 (16.2)	
Smoker	60,142 (17.0)	35 (10.7)	20 (15.4)	
Alcohol consumption				
None	215,080 (60.3)	234 (71.6)	99 (74.4)	<0.01
1–2 times/week	91,879 (25.7)	47 (14.4)	20 (15.0)	
3–4 times/week	32,086 (9.0)	21 (6.4)	8 (6.0)	
≥5 times/week	17,876 (5.0)	25 (7.7)	6 (4.5)	
Physical activity				
None	211,833 (59.3)	258 (79.1)	101 (76.5)	<0.01
1–2 times/week	78,179 (21.9)	36 (11.0)	20 (15.2)	
3–4 times/week	39,323 (11.0)	7 (2.2)	3 (2.3)	
≥5 times/week	28,044 (7.9)	25 (7.7)	8 (6.1)	
History of stroke				
No	253,662 (98.2)	251 (93.7)	97 (93.3)	<0.01
Yes	4638 (1.8)	17 (6.3)	7 (6.7)	
History of heart disease				
No	246,268 (95.3)	246 (92.1)	94 (90.4)	<0.01
Yes	12,137 (4.7)	21 (7.9)	10 (9.6)	
History of hypertension				
No	164,856 (63.5)	133 (49.4)	49 (46.7)	<0.01
Yes	94,633 (36.5)	136 (50.6)	56 (53.3)	
History of type 2 diabetes				
No	227,279 (87.9)	212 (79.1)	82 (78.1)	<0.01
Yes	31,401 (12.1)	56 (20.9)	23 (21.9)	
History of dyslipidemia				
No	242,507 (93.8)	251 (94.0)	98 (92.5)	0.84
Yes	16,087 (6.2)	16 (6.0)	8 (7.6)	
History of other diseases including cancer				
No	229,876 (88.9)	219 (82.0)	88 (84.6)	<0.01
Yes	28,576 (11.1)	48 (18.0)	16 (15.4)	
Age (years)	59.0 ± 8.8	72.5 ± 8.8	68.9 ± 9.2	<0.01
Waist circumference (cm)	82.0 ± 8.2	83.0 ± 8.9	82.9 ± 8.5	0.05
Systolic blood pressure (mmHg)	125.3 ± 15.2	130.5 ± 16.3	129.1 ± 16.3	<0.01
Diastolic blood pressure (mmHg)	77.6 ± 9.9	78.6 ± 9.9	77.9 ± 10.8	0.18
Fasting serum glucose level (mg/dL)	101.2 ± 25.3	106.7 ± 32.1	104.4 ± 26.2	<0.01
Serum creatinine level (mg/dL)	1.1 ± 1.2	1.1 ± 1.0	1.0 ± 1.0	0.94
AST level (U/L)	26.5 ± 18.4	25.9 ± 10.0	27.4 ± 14.3	0.71
ALT level (U/L)	25.1 ± 19.1	21.9 ± 12.1	23.2 ± 15.6	<0.01
γ-GT level (U/L)	38.2 ± 53.6	30.9 ± 23.8	36.1 ± 36.6	0.04

*Note.* ALT = alanine aminotransferase; AST = aspartate aminotransferase; γ-GT = gamma glutamyltransferase. Values are presented as *n* (%) for categorical variables and mean ± standard deviation for continuous variables. ^a^ *p*-values were estimated by Chi-square test for categorical variables and ANOVA test for continuous variables.

**Table 2:** Associations between visual impairment and mortality among the Korean national health examinees.

Visual Function	No. of Deaths	HR	95% CI	*p*-Value for Trend
Analyses not adjusted for physical activity ^a^				
All-cause mortality				
No visual impairment	8211	Ref.	Ref.	0.03
Mild visual impairment	34	1.17	0.81, 1.69	
Severe visual impairment	16	1.90	1.08, 3.35	
Mortality due to cardiovascular diseases				
No visual impairment	1202	Ref.	Ref.	0.02
Mild visual impairment	6	1.22	0.85, 1.76	
Severe visual impairment	5	1.84	1.04, 3.23	
Mortality due to cancers				
No visual impairment	3415	Ref.	Ref.	0.34
Mild visual impairment	9	0.83	0.39, 1.74	
Severe visual impairment	2	0.44	0.06, 3.10	
Mortality due to other diseases				
No visual impairment	3504	Ref.	Ref.	0.01
Mild visual impairment	19	1.60	1.00, 2.54	
Severe visual impairment	9	2.20	0.99, 4.90	
Analyses further adjusted for physical activity^b^				
All-cause mortality				
No visual impairment	8211	Ref.	Ref.	0.03
Mild visual impairment	34	1.16	0.81, 1.67	
Severe visual impairment	16	1.87	1.06, 3.29	
Mortality due to cardiovascular diseases				
No visual impairment	1202	Ref.	Ref.	0.03
Mild visual impairment	6	1.21	0.84, 1.75	
Severe visual impairment	5	1.80	1.02, 3.18	
Mortality due to cancers				
No visual impairment	3415	Ref.	Ref.	0.33
Mild visual impairment	9	0.83	0.39, 1.74	
Severe visual impairment	2	0.43	0.06, 3.07	
Mortality due to other diseases				
No visual impairment	3504	Ref.	Ref.	0.01
Mild visual impairment	19	1.58	0.99, 2.51	
Severe visual impairment	9	2.15	0.96, 4.79	

*Note.* CI = confidence interval; HR = hazard ratio; Ref. = reference. ^a^ Adjusted for age, sex, household income decile, residing province, smoking status, alcohol consumption, body mass index, waist circumference, systolic and diastolic blood pressure, serum levels of fasting glucose, creatinine, aspartate aminotransferase, alanine aminotransferase, and gamma glutamyltransferase, and history of stroke, heart disease, hypertension, type 2 diabetes, dyslipidemia, and other diseases including cancer. ^b^ Adjusted for the aforementioned covariates and physical activity.

**Table 3:** Associations^a^ between visual impairment and all-cause mortality stratified by physical activity.

Visual Function	No. of Death	HR	95% CI	*p*-Value for Trend
Not engaging in physical activity (None)				
No visual impairment	5921	Ref.	Ref.	0.01
Mild visual impairment	31	1.28	0.88, 1.86	
Severe visual impairment	15	1.96	1.09, 3.55	
Engaging in physical activity (≥once/week)				
No visual impairment	2234	Ref.	Ref.	0.41
Mild visual impairment	3	0.29	0.04, 2.09	
Severe visual impairment	1	1.11	0.16, 7.88	

*Note.* CI = confidence interval; HR = hazard ratio; Ref. = reference. ^a^ Adjusted for age, sex, household income decile, residing province, smoking status, alcohol consumption, body mass index, waist circumference, systolic and diastolic blood pressure, serum levels of fasting glucose, creatinine, aspartate aminotransferase, alanine aminotransferase, and gamma glutamyltransferase, and history of stroke, heart disease, hypertension, type 2 diabetes, dyslipidemia, and other diseases including cancer.

## Supplementary Materials

The Supplementary Materials are available online at https://www.mdpi.com/1660-4601/16/22/4386/s1, Methods: Assumed causal pathway and adjusted covariates, Table S1. Associations of visual impairment with all-cause and cause-specific mortality among the Korean national health examinees, excluding individuals diagnosed with vision-threatening conditions among those classified in the no visual impairment group, Table S2. Associations of visual impairment with all-cause and cause-specific mortality among the Korean national health examinees, including individuals with physical, auditory, language, intellectual, and mental disabilities, brain lesions, and other types of disability. Figure S1. Kaplan-Meier curves of survival by visual function.
