# Supplementary material for: Modification of the Association between Visual Impairment and Mortality by Physical Activity: A Cohort Study among the Korean National Health Examinees
Source: Int J Environ Res Public Health. 2019 Nov 10;16(22):4386. doi: 10.3390/ijerph16224386 (PMC6888179; doi:10.3390/ijerph16224386)
Supplement: Supplementary file 1 [file ijerph-16-04386-s001.pdf]

International Journal of
*Environmental Research and Public Health*

MDPI

*Supplementary Material*

# Modification of the Association between Visual Impairment and Mortality by Physical Activity: A Cohort Study among the Korean National Health Examinees

**Kyoung-Nam Kim, Sang Jun Park, Woosung Kim, Jungmin Joo, Haebin Kim, Kyae Hyung Kim, Ji Hoon Sohn and Yong Jin Kwon**

**Table of Contents:**

**Methods: Assumed Causal Pathway and Adjusted Covariates**

**Table S1.** Associations of visual impairment with all-cause and cause-specific mortality among the Korean national health examinees, further adjusted for physical activity.

**Table S2.** Associations of visual impairment with all-cause and cause-specific mortality among the Korean national health examinees, excluding individuals diagnosed with vision-threatening conditions among those classified in the no visual impairment group.

**Table S3.** Associations of visual impairment with all-cause and cause-specific mortality among the Korean national health examinees, including individuals with physical, auditory, language, intellectual, and mental disabilities, brain lesions, and other types of disability.

## Methods

*Assumed Causal Pathway and Adjusted Covariates*

According to the assumed causal pathways, the covariates adjusted in the main model were age (year), sex, household income (deciles, 0–10), residing province, smoking status (non-smoker, ex-smoker, or smoker), alcohol consumption (none, 1–2, 3–4, or ≥5 times/week), body mass index (<18.5, 18.5–22.9, 23–24.9, or ≥25 kg/m$^2$), waist circumference (cm), systolic and diastolic blood pressure (mmHg), serum levels of fasting glucose (mg/dL), creatinine (mg/dL), aspartate aminotransferase (U/L), alanine aminotransferase (U/L), and gamma glutamyltransferase (U/L), and history of stroke (yes or no), heart disease (yes or no), hypertension (yes or no), type 2 diabetes (yes or no), dyslipidemia (yes or no), and other diseases including cancer (yes or no).

**Table S1.** Associations [a] of visual impairment with all-cause and cause-specific mortality among the Korean national health examinees, further adjusted for physical activity.

| Visual function | No. of deaths | HR | 95% CI | *p*-value for trend |
|---|---|---|---|---|
| | All-cause mortality | | | |
| No visual impairment | 8211 | Ref. | Ref. | 0.03 |
| Mild visual impairment | 34 | 1.16 | 0.81, 1.67 | |
| Severe visual impairment | 16 | 1.87 | 1.06, 3.29 | |
| | Mortality due to cardiovascular diseases | | | |
| No visual impairment | 1202 | Ref. | Ref. | 0.03 |
| Mild visual impairment | 6 | 1.21 | 0.84, 1.75 | |

| | | | | |
|---|---|---|---|---|
| Severe visual impairment | 5 | 1.80 | 1.02, 3.18 | |
| | Mortality due to cancers | | | |
| No visual impairment | 3415 | Ref. | Ref. | 0.33 |
| Mild visual impairment | 9 | 0.83 | 0.39, 1.74 | |
| Severe visual impairment | 2 | 0.43 | 0.06, 3.07 | |
| | Mortality due to other diseases | | | |
| No visual impairment | 3504 | Ref. | Ref. | 0.01 |
| Mild visual impairment | 19 | 1.58 | 0.99, 2.51 | |
| Severe visual impairment | 9 | 2.15 | 0.96, 4.79 | |

*Note.* CI = confidence interval; HR = hazard ratio; Ref. = reference. [a]Adjusted for age, sex, household income decile, residing province, smoking status, alcohol consumption, physical activity, body mass index, waist circumference, systolic and diastolic blood pressure, serum levels of fasting glucose, creatinine, aspartate aminotransferase, alanine aminotransferase, and gamma glutamyltransferase, and history of stroke, heart disease, hypertension, type 2 diabetes, dyslipidemia, and other diseases including cancer.

**Table S2.** Associations[a] of visual impairment with all-cause and cause-specific mortality among the Korean national health examinees, excluding individuals diagnosed with vision-threatening conditions among those classified in the no visual impairment group.

| Visual function | No. of deaths | HR | 95% CI | *p*-value for trend |
|---|---|---|---|---|
| | All-cause mortality | | | |
| No visual impairment | 7250 | Ref. | Ref. | 0.02 |
| Mild visual impairment | 34 | 1.18 | 0.82, 1.70 | |
| Severe visual impairment | 16 | 1.93 | 1.09, 3.40 | |
| | Mortality due to cardiovascular diseases | | | |
| No visual impairment | 1058 | Ref. | Ref. | 0.02 |
| Mild visual impairment | 6 | 1.23 | 0.86, 1.78 | |
| Severe visual impairment | 5 | 1.86 | 1.05, 3.28 | |
| | Mortality due to cancers | | | |
| No visual impairment | 3060 | Ref. | Ref. | 0.34 |
| Mild visual impairment | 9 | 0.83 | 0.39, 1.74 | |
| Severe visual impairment | 2 | 0.44 | 0.06, 3.10 | |
| | Mortality due to other diseases | | | |
| No visual impairment | 3132 | Ref. | Ref. | 0.004 |
| Mild visual impairment | 19 | 1.63 | 1.02, 2.60 | |
| Severe visual impairment | 9 | 2.25 | 1.01, 5.03 | |

*Note.* CI = confidence interval; HR = hazard ratio; Ref. = reference. [a]Adjusted for age, sex, household income decile, residing province, smoking status, alcohol consumption, physical activity, body mass index, waist circumference, systolic and diastolic blood pressure, serum levels of fasting glucose, creatinine, aspartate aminotransferase, alanine aminotransferase, and gamma glutamyltransferase, and history of stroke, heart disease, hypertension, type 2 diabetes, dyslipidemia, and other diseases including cancer.

**Table S3.** Associations[a] of visual impairment with all-cause and cause-specific mortality among the Korean national health examinees, including individuals with physical, auditory, language, intellectual, and mental disabilities, brain lesions, and other types of disability.

| Visual function | No. of deaths | HR | 95% CI | *p*-value for trend |
|---|---|---|---|---|
| All-cause mortality | | | | |
| No visual impairment | 8554 | Ref. | Ref. | 0.04 |
| Mild visual impairment | 34 | 1.15 | 0.79, 1.65 | |
| Severe visual impairment | 16 | 1.86 | 1.06, 3.28 | |
| Mortality due to cardiovascular diseases | | | | |
| No visual impairment | 1261 | Ref. | Ref. | 0.03 |
| Mild visual impairment | 6 | 1.20 | 0.83, 1.73 | |
| Severe visual impairment | 5 | 1.80 | 1.02, 3.17 | |
| Mortality due to cancers | | | | |
| No visual impairment | 3509 | Ref. | Ref. | 0.34 |
| Mild visual impairment | 9 | 0.83 | 0.39, 1.74 | |
| Severe visual impairment | 2 | 0.44 | 0.06, 3.10 | |
| Mortality due to other diseases | | | | |
| No visual impairment | 3784 | Ref. | Ref. | 0.01 |
| Mild visual impairment | 19 | 1.53 | 0.96, 2.44 | |
| Severe visual impairment | 9 | 2.12 | 0.95, 4.72 | |

*Note.* CI = confidence interval; HR = hazard ratio; Ref. = reference. [a]Adjusted for age, sex, household income decile, residing province, smoking status, alcohol consumption, physical activity, body mass index, waist circumference, systolic and diastolic blood pressure, serum levels of fasting glucose, creatinine, aspartate aminotransferase, alanine aminotransferase, and gamma glutamyltransferase, and history of stroke, heart disease, hypertension, type 2 diabetes, dyslipidemia, and other diseases including cancer.
